# Artificial intelligence in hospitals: providing a status quo of ethical considerations in academia to guide future research

**DOI:** 10.1007/s00146-021-01239-4

**Published:** 2021-06-28

**Authors:** Milad Mirbabaie, Lennart Hofeditz, Nicholas R. J. Frick, Stefan Stieglitz

**Affiliations:** 1 Faculty of Business Administration and Economics, Paderborn University, Paderborn, Germany; 2 Professional Communication in Electronic Media / Social Media, University of Duisburg-Essen, Duisburg, Germany

**Keywords:** Artificial intelligence, Ethics, Healthcare, Hospitals, Discourse approach

## Abstract

The application of artificial intelligence (AI) in hospitals yields many advantages but also confronts healthcare with ethical questions and challenges. While various disciplines have conducted specific research on the ethical considerations of AI in hospitals, the literature still requires a holistic overview. By conducting a systematic discourse approach highlighted by expert interviews with healthcare specialists, we identified the status quo of interdisciplinary research in academia on ethical considerations and dimensions of AI in hospitals. We found 15 fundamental manuscripts by constructing a citation network for the ethical discourse, and we extracted actionable principles and their relationships. We provide an agenda to guide academia, framed under the principles of biomedical ethics. We provide an understanding of the current ethical discourse of AI in clinical environments, identify where further research is pressingly needed, and discuss additional research questions that should be addressed. We also guide practitioners to acknowledge AI-related benefits in hospitals and to understand the related ethical concerns.

**Supplementary Information:**

The online version contains supplementary material available at 10.1007/s00146-021-01239-4.

## Introduction

Ethical considerations are not limited to the philosophy discipline (e.g., Ploug and Holm 2020), but are also highly relevant in healthcare and social science-related disciplines such as information systems (IS) (e.g., Wang 2020). However, current developments in artificial intelligence (AI) give rise to profound novel ethical challenges when applied in healthcare, possibly posing a threat to patients (Jain et al. 1996; Rudin 2019; Mirbabaie et al. 2021a).

The implementation of AI recently became more distributed in hospitals worldwide (Knijnenburg and Willemsen 2016; Luger and Sellen 2016; Li et al. 2019b), creating discernible benefits assisting medical experts in hospitals (Rauschert et al. 2020; Rong et al. 2020). The term AI is usually associated with human-like behavior, but it must rather be considered as a ubiquitous concept (Siau and Wang 2018). Current applications have been developed for particular tasks (e.g., Frick et al. 2019a), such as taking advantage of medical data to generate predictions or derive recommendations (Krittanawong et al. 2017; Ku et al. 2019). For example, AI monitors patients’ health conditions to support healing and regeneration (Pereira et al. 2013) and assists physicians in diagnosing diseases (Mirbabaie et al. 2021b) and planning suitable treatments (e.g., De Ramón Fernández et al. 2019; Li et al. 2019a, b; López-Martínez et al. 2019). However, some AI approaches possess certain technical restrictions which can lead to diagnostic results not being transferable to other circumstances or not being comprehensible to humans, i.e. remaining a black box (Anderson and Anderson 2007; Menai 2015; Knight 2017; Burton et al. 2019; Devi et al. 2019). Scholars and practitioners are also concerned with preventing inequitable usage and unfair information practices (Salerno et al. 2017; Sonja et al. 2018; Libaque-Sáenz et al. 2020). Furthermore, AI still learns from medical data that is preprocessed by humans and thus might contain bias or prejudices (Kara et al. 2006; Hirschauer et al. 2015; Ploug and Holm 2020; Alami et al. 2020).

Enthusiasts claim strong reasons for the application of AI in hospitals (Ploug and Holm 2020); nevertheless, there are ominous threats possibly leading to AI becoming destructive (Arnold and Scheutz 2018). AI is a powerful but inscrutable tool unleashed with potential dubious effects for areas in which it is applied, e.g., healthcare and/or hospitals (Crawford and Calo 2016). Research on ethical considerations of AI in hospitals is no longer a mere part of science fiction but a real-world concern (Luxton 2014a, b). Despite existing studies on ethics of AI in healthcare (e.g., Alami et al. 2020; Arnold and Scheutz 2018; Ploug and Holm 2020), we argue that current research does not consider the growing significance of the topic in a diversified enough manner, but is rather narrowly focused on traditional explorations.

The current ethical discourse on AI is rather limited and usually presented in an unsystematic manner while also being conducted in separate disciplines (Brendel et al. 2021). There should instead be an increasing debate about ethical concerns (Porra et al. 2020) taking into account the multiple characteristics, principles, and dimensions of AI. Thus, our study follows a more holistic approach by identifying fundamental literature and pioneering works from diversified research domains. We aim to summarize ethical considerations into a research agenda for academia. Precisely, we intend to encourage the discourse on ethical considerations of AI in hospitals from an interdisciplinary perspective. We argue that this is of great interest to researchers and practitioners because the application of AI in hospitals is expected to increase heavily over the next decade and the impact on healthcare could be significant (Mirbabaie et al. 2021a).

Physicians still consider AI to be simple programs, tools, or algorithms that provide support in executing a certain task but they do not recognize (or even ignore) the fact that AI is capable of continuously learning and developing over time (Mitchell et al. 2018) and that it acts independently while delivering superior results compared to humans. There is an urgent demand for interdisciplinary research to comprehend the ongoing discourse on ethical considerations and dimensions of AI in hospitals and to understand the intricacies of this ever-evolving research area. By providing a holistic picture of ethical considerations and dimensions on AI in hospitals that are currently being researched, we aim to capture the current status quo and to guide pertinent future research directions. To address this urgent issue, our research is guided by the following research questions:

RQ1: *What is the current discourse in academia and what are opinions of physicians regarding ethical considerations and dimensions of artificial intelligence in hospitals?*

RQ2: *What are future directions for interdisciplinary ethical research on artificial intelligence in hospitals?*

We followed a modified discourse approach following the suggestions of Larsen et al. (2019) and identified as well as analyzed the domain ecosystem of ethical considerations and dimensions of AI in hospitals for a corpus construction. We thus performed descriptive research examining existing literature that describes the current situation (Bell 1989; Bear and Knobe 2016). In addition, we conducted semi-structured interviews with domain experts to further elaborate on and highlight related ethical challenges of AI in the clinical environment. This prescriptive approach contains implications and consequences as well as future recommendations (Bell 1989; Bear and Knobe 2016).

This paper contributes to theory by summarizing and structuring the status quo of recent research on ethical considerations and dimensions of AI in hospitals. Researchers will find the overview helpful to understand the current ethical discourse of AI in a hospital setting. To assist future investigations, we outline ethical constructs on AI in hospitals with which recent research is concerned. Furthermore, we outline an agenda explaining where further research is pressingly needed, and which questions need to be addressed. Practitioners will comprehend the differences between currently applied systems in hospitals and recent AI developments. Furthermore, medical specialists will be able to understand the extent to which AI is beneficial for clinical settings and the ways in which the stakeholders involved, i.e. physicians and patients, can benefit from its implementation. In terms of implications for society, readers will realize that AI is already used in hospitals and that its distribution continues to grow. Individuals will further understand that multiple issues regarding the application of AI in hospitals remain unaddressed.

## Literature background

In this section, we start by explaining the concept of AI, followed by outlining illustrative examples of applications in hospitals. We then describe current ethical principles in healthcare, and finally, we illustrate ethical considerations associated with AI in hospitals.

### AI applications in hospitals

Hospitals face a variety of issues that reduce the quality of care such as delayed patient flow or erroneous surgery scheduling (Ker et al. 2018; Bygstad et al. 2020). The introduction of AI might improve these types of common issues and yield sustainable advantages. This explains why medical research and practice are increasingly concerned with possible applications of AI (e.g., Bargshady et al. 2020; Jiang et al. 2017; Rauschert et al. 2020). AI is not a specific technology that is granted to a single discipline, but rather a collection of several concepts that constantly evolve (Barredo Arrieta et al. 2020). AI can generally be defined as “the ability of a machine to perform cognitive functions that we associate with human minds, such as perceiving, reasoning, learning, interacting with the environment, problem-solving, decision-making, and even demonstrating creativity” (Rai et al. 2019, p. iii). Simply put, AI aims to imitate human-like behavior (Krittanawong et al. 2017); however, current implementations are still far from achieving this goal (Brachten et al. 2020).

Applications of AI are rather narrowed down to a specific task (Batin et al. 2017; Frick et al. 2019b; Mirbabaie et al. 2020) but commonly generate superior results compared to humans. When integrated into the existing technical infrastructure of hospitals, AI accelerates data collection from multiple sources (Nasirian et al. 2017), provides medical experts with more accurate and timely information (Atherton et al. 2013; Preece et al. 2017; Diederich et al. 2019), tailors to the needs of patients and their treatment processes (Dilsizian and Siegel 2014) and enhances integration with other hospital IS (Serrano et al. 2020). AI continuously learns and develops over time by processing various types of medical information from multiple years of experience using divergent data sources (Mitchell et al. 2018). Conclusions are based on a larger sample size compared to those of medical professionals (Neill 2013) and AI is more likely to provide objective decisions. AI is also more likely to evaluate patients’ conditions based on medical facts, as their systems do not rely on subjective impression, situations, emotions, or time of the day (Gnewuch et al. 2017; Seeber et al. 2020).

AI already supports multiple processes within hospitals. For example, AI guides patients with exercise promotion, medication adherence (Bickmore et al. 2010; King et al. 2013), chronic disease self-care management (Kimani et al. 2016), and daily diabetes routines (Shaked 2017) as well as accelerating the gathering of medical information in preparation for therapy and forwarding them to physicians (Denecke et al. 2018). In these examples, patients use AI in the form of a conversational agent (CA), intelligent systems that interact with and augment humans’ abilities (Mirbabaie 2021). Interacting with CAs not only assists patients but also clinicians in the treatment of certain diseases.

AI also assists medical experts within disease diagnostics such as ectopic pregnancies (De Ramón Fernández et al. 2019), neonatal sepsis (López-Martínez et al. 2019), or coronary artery disease (Li et al. 2019a). Medical data are thereby processed, evaluated, and classified using AI algorithms to estimate probabilities and enable clinicians to detect diseases earlier, thus allowing them to treat patients more effectively. The implementation of information technologies such as AI can impact hospitals’ revenue cycle management and consequent financial sustainability (Singh et al. 2021).

Even though existing endeavors provide justification for the use of AI in clinical environments, researchers and practitioners are frequently confronted with ethical questions eventually preventing possible applications due to the fear of causing unpredictable harm to patients. The discussion on autonomous driving showed that the expectations on AI can be even higher than towards human. The same could apply for the use of AI in hospitals and therefore need further examination.

### Ethical principles in healthcare

Ethics is an interdisciplinary field of study and a complex concept that governs the accumulation and interplay of moral principles (Siau and Wang 2020). Moral principles describe norms for the behavior and actions of groups or individuals in a society (Nalini 2019) that guide entities (such as humans or intelligent robots) regarding what is right and wrong. Overall, it is tough to determine where ethical behaviors begin and where unethical behavior comes into play. As one approach to determine what is right and wrong, virtues can be considered. Virtue ethics is part of normative ethics and addresses the principles in which individuals believe (Siau and Wang 2020). Virtue ethics can be seen as an overarching moral principle to help make morally problematic decisions (such as which treatments should be provided in hospitals based on a diagnosis made by an AI). In this study, we therefore focused on a virtue-ethical perspective regarding AI applications in hospitals, concentrating on treatment decisions.

Research on ethical considerations in healthcare is generally divided into three fields (Page 2012): the first field focuses on ethical developments of future healthcare experts throughout their medical training (Price et al. 1998; Bore et al. 2005). The second assesses individual ethical attitudes and how they differ among medical professions (Rezler et al. 1990, 1992). The third is concerned with the evaluation of ethical principles and their applications within treatment of patients (Hebert et al. 1992; Price et al. 1998). Ethical principles in medicine can be traced back to those of the physician Hippocrates (400 BCE), on which the concept of the Hippocratic oath is rooted (Miles 2005). The Hippocratic oath was a Greek document containing ethical standards for physicians which, for example, covers protecting the privacy of patients (Fox and James 2020). Today, the majority of medical graduates swear some kind of oath that is based on the Hippocratic oath (Hulkower 2010). Since its origin, various concepts have been developed for ethical guidelines for treating patients. The principles of biomedical ethics of Beauchamp and Childress (2019) have found great acceptance in medicine. The authors define four core principles of bioethics. (1) The principle of beneficence involves the expectation that healthcare professionals act in a way that benefits patients. (2) The principle of non-maleficence aims at avoiding any harm to involved individuals, i.e., patients or physicians. (3) The principle of autonomy respects the capabilities of individuals to make independent decisions. (4) The principle of justice specifies that all patients should be treated equally (Beauchamp and Childress 2019). Treatment ethics is intentionally defined rather broadly to allow room for individual considerations and prioritizations by physicians. Besides the principles of bioethics, ongoing research and practice are increasingly shaped by associations. There are country-specific organizations like the American Medical Association (USA) or the Academy for Ethics in Medicine (Germany), which define standards for honorable behavior of physicians when treating patients and encourage the scientific discourse on ethical questions in medicine (Riddick 2003; AEM 2020; AMA 2020). Furthermore, there are overarching institutions like the European Council of Medical Orders (CEOM 2020), which promote the practice of high-quality medicine in light of the patients’ needs.

Despite the existence of ethical guidelines and principles for medical professionals, the entire healthcare system is regularly confronted with new ethical considerations. A recent example from Poland demonstrates that local governments affect healthcare and affect the majority of a population. The country’s constitutional court declared abortions of children with malformations to be illegal (Amnesty International 2020). Besides restricting the freedom of choice of expectant parents, practicing physicians are restrained by this law and must abide even when an alternative decision might be more appropriate. Human rights activists and the Polish opposition heavily criticized the ruling of the constitutional court, arguing that illegal abortions will rise (Walker 2020). Another example of ethical considerations is the current discussion on distributing a potential COVID-19 vaccine. In principle, it seems reasonable that vaccinations should be given in a sequence based on profession. It is suggested, for example, that people in caring jobs should receive preferential treatment. Naturally, the question arises which professions within care should be prioritized, e.g., nursing, or elderly care?

The examples presented are intended to illustrate the idea that ethical principles are not only established by medical workers but are also heavily impacted by external forces. Likewise, AI applied in healthcare needs to adjust to a continuously changing environment with frequent interruptions (Wears and Berg 2005; Menschner et al. 2011; Rosen et al. 2018), while maintaining ethical principles to ensure the well-being of patients. Thus, in our study, we use the four core principles of biomedical ethics as suggested by Beauchamp and Childress (2019) as a conceptual categorization to classify our findings. This is then used to provide a research agenda for academia to examine the ethical challenges of using AI in hospitals.

### Ethical considerations of AI in hospitals

Recent AI implementations in hospitals and in healthcare in general come with a variety of ethical considerations. For example, AI is associated with bias, discrimination, opacity, and rational concerns and intentions (e.g., Arnold and Scheutz 2018; Gruson et al. 2019; Ploug and Holm 2020) as much as it is associated with transparency, trust, responsibility, liability, and explainability (e.g., Alami et al. 2020; Wang 2020). A recent study by Ploug and Holm (2020) investigated the ethical concerns of AI for medical diagnostics and treatment planning. The authors argued that patients should be able to withdraw from being evaluated by AI because a trustful relationship between physicians and patients is essential for the success of the treatment process. Furthermore, Ploug and Holm (2020) explain that there are problems regarding bias and opacity for the patient, related implications for the entire healthcare sector, and rational concerns about impacts on society. Another study by Alami et al. (2020) provides a synthesis of key challenges posed by AI. Besides technological, organizational, and economic issues, the authors also raise several ethical obstacles. For example, AI applications can be distinguished between decision-support tools and decision-making tools. AI as decision-support tools assist medical specialists with specific tasks, e.g., within the diagnostic process (e.g., De Ramón Fernández et al. 2019; López-Martínez et al. 2019). When applied as a decision-making tool, AI will derive conclusions on its own without being supervised by physicians. However, it is yet to be defined who is held responsible for AI-based decisions leading to errors in the treatment process. Another issue illustrated by Alami et al. (2020) is the potential unexplainability of algorithmic outcomes, i.e. black box, posing a high risk to patients’ well-being (Knight 2017; Rudin 2019). Of course, this makes it nearly impossible to build trust in the AI’s decisions, especially when patients’ lives are at stake.

Compared to ethical guidelines in healthcare, there are neither standardized regulations for the application of AI in healthcare nor in hospitals. However, most healthcare systems acknowledge the rapid development of AI for medical purposes (Duan et al. 2019) causing organizations and governments to define relevant ethical frameworks. For example, the European Union has developed the “European Ethics Guidelines for Trustworthy AI” defining its recommendations for trustworthy AI and key requirements for safety and for societal and environmental well-being (EU 2020). Furthermore, the World Health Organization has explained ethical challenges for the “global development and implementation of artificial intelligence systems in healthcare” (Bærøe et al. 2020, p. 261) and continually proposes suggestions for the ethical development and usage of AI. Besides global observations of AI within healthcare, research is equally concerned with deriving ethical principles, guidelines, and frameworks. For example, Floridi et al. (2018) developed an ethical framework for a good AI society based on the four core principles of bioethics of Beauchamp and Childress (2019). The authors added a fifth dimension explicability explaining the “need to understand and hold to account the decision-making processes of AI” (Floridi et al. 2018, p. 700).

Since the authors took an initial approach to tackle ethical issues regarding AI, we extended our conceptual categorization to include the principles of biomedical ethics of Beauchamp and Childress (2019) as well as the dimension of explicability (Floridi et al. 2018) which in most research is interchangeably used for explainability. We used these two pieces of work as the foundation of this work because they have been frequently cited and are centrally concerned with ethical dimensions of AI in various domains. Additionally, we used these frameworks because one includes a clear philosophical perspective on virtue ethics and both a bioethical perspective that is applicable to treatment ethics and the context of healthcare. Even though these articles did not focus on healthcare or hospitals themselves, the discussed ethical principles have been frequently used in other articles. Despite increasing studies being conducted on ethical considerations, current approaches are mostly congruent or very alike and focus on one specific discipline or a certain abstraction level. We thus argue that future endeavors would benefit from an alternative discourse from an interdisciplinary perspective that guides pertinent research directions.

## Research design

Ethical discourses on the impact of new technologies are usually very unsystematic, as there is often no fundamental manuscript on which to base them. Although there have been some pioneering works, which are often quoted, many parallel discourses emerge, which make little reference to each other. In addition, ethical discourses are usually conducted separately in certain disciplines. To investigate how academia can contribute to the responsible use of AI in digital health and practical health in hospitals, we identified fundamental manuscripts following adapted version of the discourse approach proposed by Larsen et al (2019). Based on this, we identified ethical principles and their relationships and highlighted these via expert interviews with hospitals physicians and other decision-makers in hospitals.

### Modified discourse approach

For systematic literature analysis, new approaches are constantly being developed (vom Brocke et al. 2009, 2015). However, with the increasing number of publications, it is becoming more and more difficult to find a method that can provide a comprehensive picture of a discourse. The discourse approach is an instrument that creates a citation network based on fundamental manuscripts of a theory, a model, a framework, or a research domain (Larsen et al. 2019). It starts with the identification of fundamental theory-building papers (L1), followed by theory-contributing and other papers that cite these L1 papers (L2). In a last step, papers are identified by means of citations, which influenced the L2 papers (L3). Larsen et al. (2019) call the sum of these L1, L2, and L3 papers “the theory ecosystem.”

**Fig. 1:**
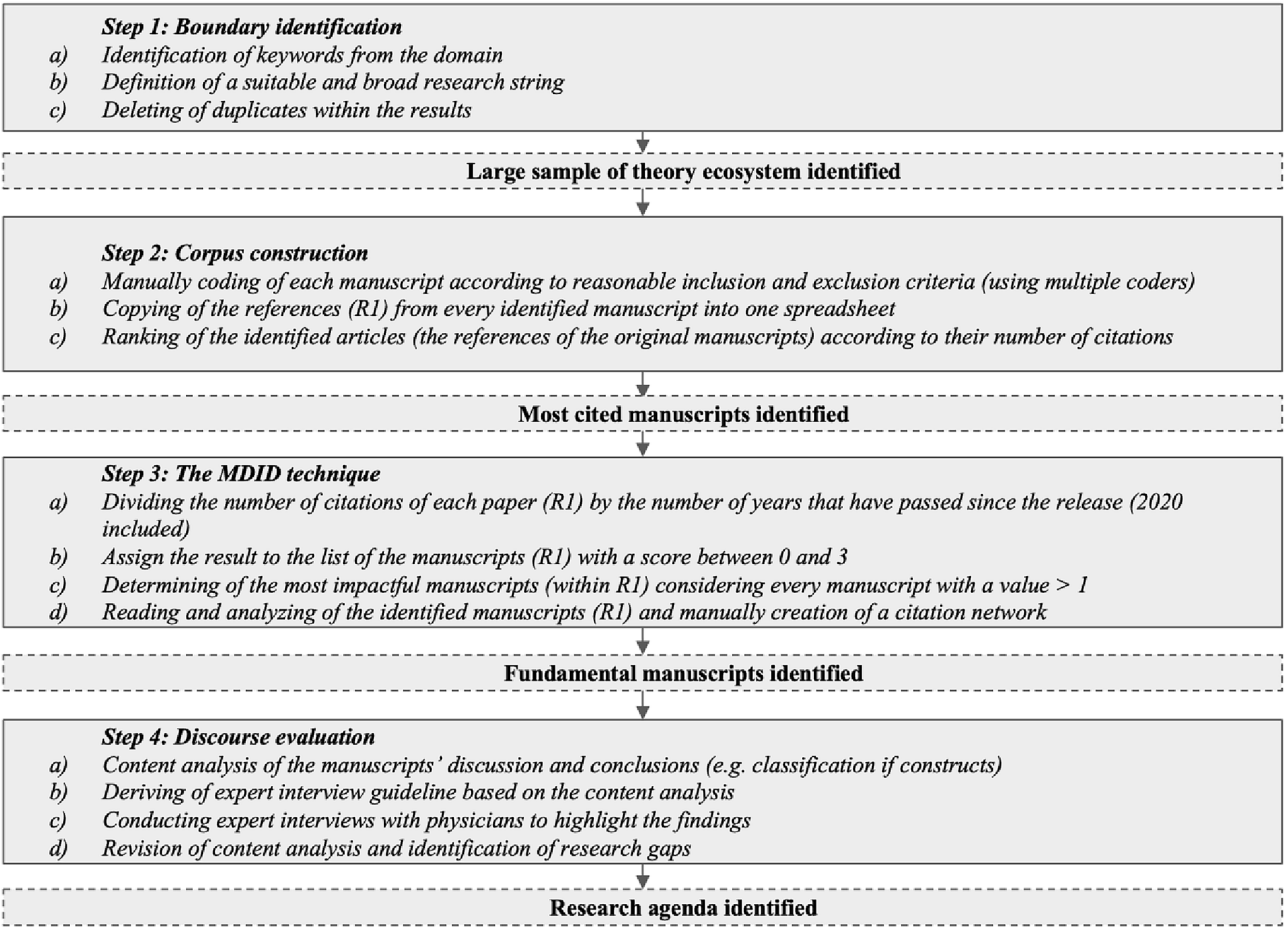
Adapted discourse approach based on Larsen et al. (2019) to derive a research agenda

However, it is not always obvious which manuscripts form the fundamental basis for a discourse. The discourse on the responsible use of AI in hospitals is a rather new one, as fundamental manuscripts have yet to emerge. Therefore, the discourse approach cannot always be applied exactly according to Larsen et al. (2019). We therefore propose a modified discourse approach. The aim of our approach is to start vice versa by identifying fundamental L1 manuscripts and to derive a research agenda for ethical considerations of AI in hospitals. As the IS perspective is rather interdisciplinary, we started our research to the field of IS and related disciplines using the litbaskets.io database with 3XL search. Our method consisted of four phases following the recommendations by Larsen et al. (2019) and highlighting the outcomes with interview findings. An overview of the applied research approach is provided in Fig. 1 and will be presented in the following sub-sections.

#### Boundary identification

A research domain is less a set of characteristics and more an evolving discourse between scholars (Larsen et al. 2019). To reflect this discourse, a starting point is first required. According to Larsen et al. (2019), this initial point is the origin of a theory, framework, or model. In this paper, however, we wanted to identify the status quo in research on ethical considerations on AI use in hospitals. Therefore, we based our boundary identification on elements of other systematic literature reviews such as a comprehensive keyword search as proposed by vom Brocke et al. (2015). To identify a first sample in the theory ecosystem, we first collected frequent keywords related to ethical principles of using AI in health and especially in hospitals. We selected artificial intelligence as the keywords as well as related terms that focus on more anthropomorphic forms of AI, because our focus was on technology that is also perceived as an AI by both the physicians and the patients. In addition, we selected ethic* and moral* as relevant keywords because they most precisely represented what we wanted to examine from a philosophical point of view. Furthermore, we selected common terms from the area of digital health. Afterwards we formulated a broad and comprehensive search string including the following terms:

(AI or “artificial intelligence” or “chatbot*” or “chat-bot*” or “conversational agent*” or “digital assistant*” or “virtual assistant*” or “personal assistant*” or “virtual agent*” or “ai-based system*”) AND (health or "health care" or healthcare or “digital health” or “hospital*” or medicine or medical) AND (“ethic*” or “moral*”)

We applied the search string on Scopus and used litbaskets.io (3XL search) to receive an interdisciplinary focused sample of manuscripts (Boell and Blair 2019). In addition, we manually searched for high-ranked conference articles (in International Conferences on Information Systems, European Conference on Information Systems, Hawaii International Conference on Systems Sciences, Americas Conference on Information Systems, Pacific Conference on Information Systems, Australasian Conference on Information Systems, and the German Wirtschaftsinformatik). In our initial sample, we focused on IS publications since our aim was to visualize and reflect the interdisciplinary discourse. However, as a basic search is not capable of providing a holistic overview, and we were also interested in retrieving literature outside the IS discipline, we conducted both a backward and forward search. In the backward search, we gathered the reference lists in the bibliographies of all the papers from the initial search and assessed their relevance regarding our research goal. Within the forward search, we considered every paper identified in the previous steps and analyzed literature that cited these identified papers after their initial publication. We thus expanded our search to other scientific domains and outlets. For example, we identified publications from healthcare (e.g., Journal of the American Medical Association) and philosophy (e.g., Philosophical Transactions of the Royal Society).

We conducted our literature search between September and October of 2020. After removing duplicates from the results, we identified 104 manuscripts as our initial sample. This sample consisted of interdisciplinary journals and high-level conference articles and was labeled as potential L2 articles (Larsen et al. 2019) who cite the fundamental manuscripts of the discourse on the ethical use of AI in healthcare.

#### Corpus construction

As a next step, we investigated the identified literature in more detail. Our aim was to understand the discourse on the ethical dimensions of AI in healthcare and especially in hospitals. We, therefore, manually scanned the 104 identified manuscripts according to their topic relevance. We excluded papers that did not directly address ethical dimensions and articles that did not address AI or AI-related technologies. We included manuscripts that covered both ethics and AI. Two experienced coders created a codebook and applied the exclusion and inclusion criteria to the manuscripts from the first search, following a title, abstract, and keyword scan method. This led us to 60 manuscripts that we considered the most relevant for the ethical discourse on AI in healthcare.

However, we knew that not all relevant articles for a discourse can be identified by a keyword search (Larsen et al. 2019). If a keyword search is too broad, it can lead to a list containing far more manuscripts than is practical to read; and if a keyword search is too narrow, that can result in missing highly relevant articles. To address these issues, we copied all references from these 60 manuscripts into one list, which led us to 2433 references. As our aim was to identify fundamental manuscripts for the ethical discourse on AI in healthcare, we ranked those references according to how often they were cited in the initially identified papers. The number of citations per paper within the list of all references is shown in Table 4 in the "Appendix".

#### Identification of fundamental manuscripts for the ethical discourse on AI in healthcare

Although the number of citations is an important indicator to measure the relevance of a manuscript within a discourse (Larsen et al. 2019), the time span between the publications also needs to be considered. To take publication time spans into account, we propose a manual detection of implicit domain (MDID) technique. We divided the number of citations of each paper by the number of years that have passed since the date of publication. This resulted in a score between 0 and 3 citations per year within the identified corpus. This score does not represent the overall citations per year of the manuscripts, but rather the number of times they were cited per year within the 60 papers that we identified as relevant for the ethical discourse on AI in healthcare. Among those, a few papers had a score > 1 and most of the papers scored lower than 1. The score describes the impact and relevance of the manuscript on the current discourse on AI in healthcare. To better understand the distribution of the scores, we visualized the dissemination of the scores in a graph. We found that there was a small group of manuscripts that stood out and scored higher than the majority of the articles. We identified these papers due to the visible threshold in the graph. This small group of papers scored 1.3 or higher and consisted of only 15 manuscripts. In addition, we manually scanned how these manuscripts were cited within the identified corpus of 60 papers to ensure that they were not only mentioned as a side note. We considered all of these 15 manuscripts as the fundamental articles. Additionally, these 15 manuscripts came closest to what Larsen et al. (2019) had described as L1 manuscripts. Those manuscripts are listed in Table 1.

**Table 1:** Identified fundamental manuscripts of the discourse on the ethical use of AI in healthcar**e**

Authors	Count	Score
Vayena et al. (2018)	8	2.667
Ting et al. (2017)	8	2
Char et al. (2018)	6	2
McKinney et al. (2020)	2	2
Zeng et al. (2019)	2	2
Yu et al. (2018)	5	1.667
Gulshan et al. (2016)	8	1.6
Reddy et al. (2019)	3	1.5
Yu and Kohane (2019)	3	1.5
Schiff and Borenstein (2016)	3	1.5
Parikh et al. (2019)	3	1.5
Luxton (2019)	3	1.5
He et al. (2019)	3	1.5
Froomkin et al. (2019)	3	1.5
Cath (2018)	4	1.334

**Fig. 2:**
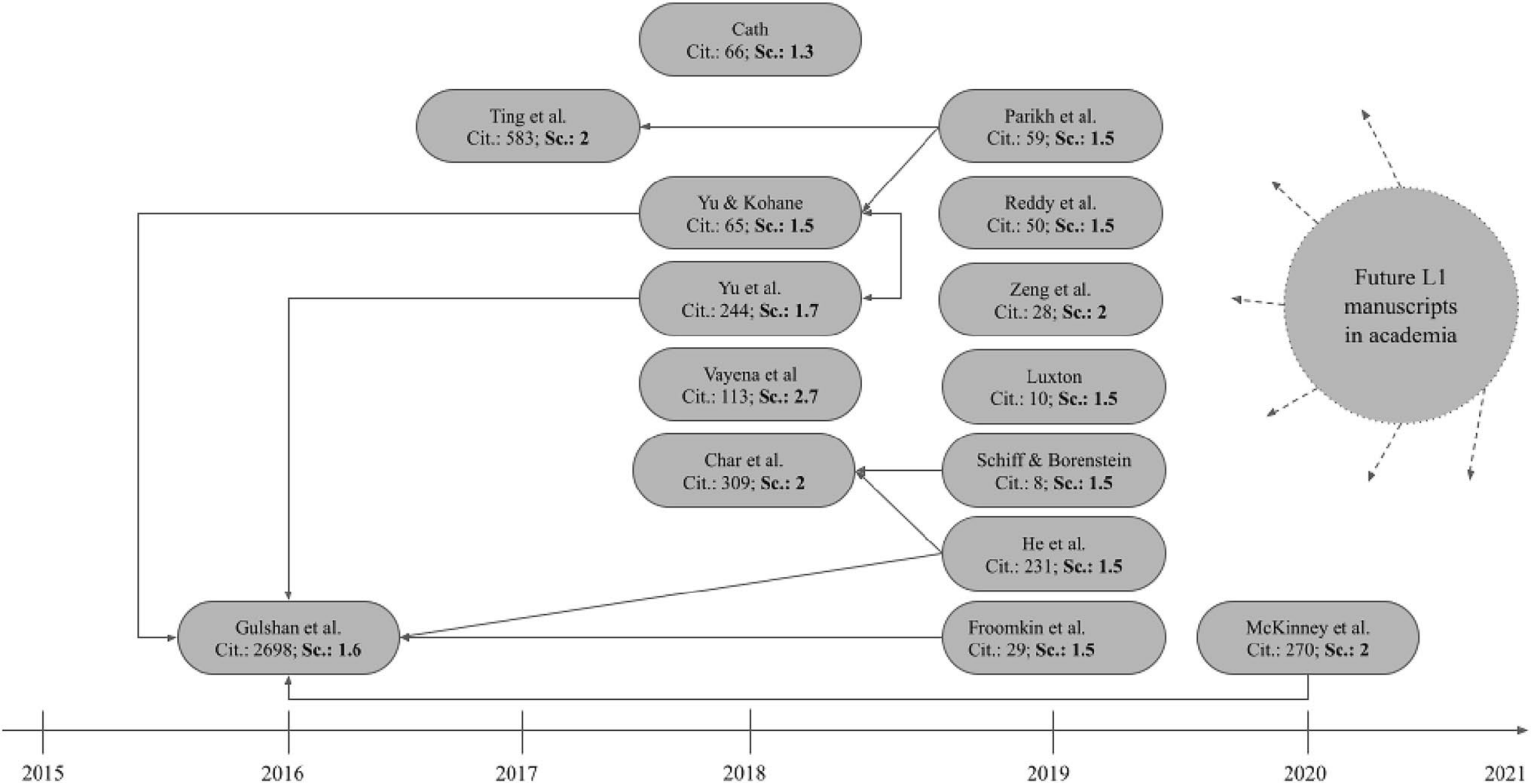
Citation network of the 15 fundamental manuscripts

As our aim was to understand and structure the ethical discourse on AI in hospitals, we further analyzed those manuscripts manually and created a citation network (Fig. 2). We scanned the manuscripts for common patterns and extracted the ethical principles for using AI in hospitals to provide a research agenda for academia.

### Expert interviews

Besides using the discourse approach as a fruitful method to obtain a comprehensive picture of the knowledge within a certain domain (Larsen et al. 2019), we also conducted semi-structured expert interviews to highlight and underpin our findings. Expert interviews preserve knowledge from individuals with advanced experience in the research domain under investigation (Meuser and Nagel 2009). We thus initially defined criteria to find appropriate participants. Since discussions on ethics in medicine are as ancient as the discipline itself, we intended to gain a holistic overview from experts of varying age groups. We further searched for medical experts working in hospital clinics who are frequently confronted with ethical questions impacting the well-being of patients. Following the recommendation of Creswell and Creswell (2018), three to ten individuals should be included for qualitative research. Moreover, we use the interviews to elaborate on and highlight our findings rather than to validate a theory. In total, we conducted six expert interviews with doctors and senior level experts in the context of hospital digitization from different medical disciplines. We interviewed one physician from obstetric care (resident doctor) and three surgeons from cranio-maxillofacial surgery (two senior physicians and one resident doctor). In addition, we spoke with a chief physician from a large hospital and a head of corporate communication with experience in digitization and change management in hospitals. An overview of our sample is outlined in Table 2. To guarantee anonymity of our interviewees, we used the synonyms E1–E6 in the following sections.

**Table 2:** Sample overview of expert interviews with physicians and senior level experts

Interviewee	Gender	Age	Tenure (years)	Position	Discipline	Hospital	Duration
E1	f	31	3.5	Resident doctor	Obstetric care	University Hospital of Frankfurt, Germany	28:17
E2	f	38	7	Senior physician	Cranio-maxillofacial surgery	University Hospital of Dusseldorf, Germany	31:38
E3	f	35	5	Senior physician	Cranio-maxillofacial surgery	University Hospital of Dusseldorf, Germany	30:30
E4	f	31	2	Resident doctor	Cranio-maxillofacial surgery	University Hospital of Dusseldorf, Germany	35:42
E5	m	67	20	Chief physician	Anesthesia	Retired	42:33
E6	m	44	17	Head of Corporate Communications	Digitization Think Tank	Clinical Center Dortmund, Germany	32:41

We used an open interview technique to provide the experts with enough room to elaborate on their subjective beliefs and experiences (Meuser and Nagel 2009). We structured the interview with a prefixed guideline (Table 6 in the "Appendix") with central questions referring to our research question (Qu and Dumay 2011). Initially, we described the interview process to the interviewee, including a short briefing of the study and the rights of the participants, followed by a verbal consent to the interview being recorded. In the first official phase, we asked general questions on the expert’s characteristics, current position, and duties within the practicing discipline. This helped us to understand the clinical environment of the expert while making the interviewee comfortable with the interview situation. The second phase served as a foundation to comprehend which ethical considerations physicians are confronted with and whether they follow a certain codex. Within the third phase, we asked question on what ethical problems technology in general might cause and how they are capable of resolving ethical issues. The fourth phase began by asking interviewees what they associate with AI. After receiving their answers, we provided a definition of AI to achieve the same level of knowledge among all participants for the remainder of the interview. We then asked specific questions about the application of AI in hospitals, e.g., how AI might support clinical processes, which factors are crucial for successful deployment, and which ethical guidelines AI must follow. In the fifth phase, the participants were asked to elaborate on future ways in which AI implementations in hospitals could improve the clinical procedures. The interview concluded by providing the interviewee with a chance to ask further questions or to provide additional information, followed by a debriefing by the interviewer.

The data were collected between September and October of 2020 by two researchers. As this period was still strongly influenced by the COVID-19 pandemic, all interviews were conducted via a virtual call. As we were not interested in the expert’s substantive statements rather than physical gestures or facial expressions, we recorded the audio and not the video signal and, respecting data privacy protection, deleted the recordings once the analysis of the interview was finished. For the examination of the retrieved data, we conducted a qualitative assessment of content analysis as previously proposed (Schilling 2006). This helped us to reduce the volume of the data by removing unnecessary words to form short and concise sentences. We paraphrased the experts’ explanations by carefully listening to each interview recording, then further generalized and reduced the contents, leading to comprehensive statements.

The analysis of the data was performed using a thematic analysis where paraphrasing was done shortly after the interviews were conducted. We derived deductive categories based on the constructs as identified from the discourse approach and used them as clusters (Glaser 2013). We thereby intended to obtain an understanding of the status quo and prospective orientations. This research approach can be classified as a descriptive-prescriptive procedure because experts described the situation, e.g., what has happened or what is happening now and what should happen in the future (Bear and Knobe 2016). Following the recommendations of (Gioia et al. 2013), we used short paragraphs or sentences as coding units, i.e. open coding. We used simple phrases or in vivo (second-order themes) to code the data, then categorized them under the constructs from the discourse approach (first-order theme). The coding process was collaboratively done by two researchers to distribute the effort of the analysis process, prevent a unilateral view of the data, and ensure intercoder reliability. Since the expert interviews were conducted with German participants working in German hospitals, the excerpts have been translated into English for the reader’s understanding.

## Results

We were able to identify 15 manuscripts that we could classify as fundamental by means of our modified discourse approach. The manuscripts were mostly published in medical journals, Nature, or Science (He et al. 2019; Parikh et al. 2019; Yu and Kohane 2019; McKinney et al. 2020). Among the papers, we found theoretical papers as well as empirical papers. Many manuscripts established principles for the ethical use of AI in hospitals or discussed different fields of application or types of AI. Although principles were strongly intertwined and we perceived some overlaps when directly comparing definitions between some papers, we could extract 18 unique ethical principles from the literature following Suddaby (2011). We consider these principles as mutually exclusive as they differed in their descriptions when comparing the 15 fundamental manuscripts. We classified the findings of our interviews into the four first-order themes beneficence, non-maleficence, justice, and autonomy and into the 18 s-order themes which represent the principles in Table 3.

**Table 3:** Ethical principles for the use of AI in hospitals extracted from the fundamental manuscripts

Type of issue	Principle	References	Description
Regulatory issues	Accountability	Cath 2018; Vayena et al. (2018), Zeng et al. (2019) and Reddy et al. (2019)	The determination of who is accountable for errors, who is socially responsible for the outcome of an AI, and which legal obligations have to be taken into account should be ensured
	Responsibility	Cath (2018), Char et al. (2018), Zeng et al. (2019) and Luxton (2019)	
	(Legal) liability	Schiff and Borenstein (2016), Vayena et al. (2018), Yu et al. (2018), Luxton (2019), and Reddy et al. (2019)	
	Privacy	Cath (2018), Vayena et al. (2018), Zeng et al. (2019) and He et al. (2019)	The protection of users’ data and the compliance with general data protection regulations should be ensured
Normative issues	Avoiding bias and harms	Cath (2018), Char et al. (2018), Parikh et al. (2019), Reddy et al. (2019) and Yu and Kohane (2019)	The prevention of damage to one or more patients from the use of AI in healthcare should be ensured
	Patient safety	He et al. (2019) and Parikh et al. (2019) and McKinney et al. (2020)	
	Fairness	Cath (2018), Vayena et al. (2018) and Zeng et al. (2019)	The avoidance of discrimination of patients should be ensured using algorithmic fairness
	Informed consent	Schiff and Borenstein (2016), Ting et al. (2017) and Froomkin et al. (2019)	It should be ensured that physicians be able to explain the exact use of an AI to be sure that the patients know to what they are consenting
Technical issues	Interoperability and generalizability	He et al. (2019), Parikh et al. (2019) and McKinney et al. (2020)	It should be ensured that the training data for an AI represents a large population to provide interoperable and generalizable systems
	Iterative controllability and updatability	Yu and Kohane (2019)	It should be ensured that AI in hospitals is always controlled by trained physicians and updated with clinical workflow disruption
	Vigilance	Yu et al. (2018)	It should be ensured that responsible physicians frequently monitor the AI system
	Security	Zeng et al. (2019)	It should be ensured that the system has a certain level of robustness against cyber-attacks
Organizational issues	Feasibility and humanity	Gulshan et al. (2016), Char et al. (2018), Yu et al. (2018), Zeng et al. (2019) and McKinney et al. (2020)	It should be determined if and how AI is capable of improving care in hospitals
	Education of an AI-literate workforce	He et al. (2019)	It should be ensured that healthcare professionals are well trained and educated in the fields of medical informatics and statistics
	Interventions	Parikh et al. (2019)	It should be ensured that the output of a predictive AI is accompanied by guidance for medical interventions
	Explainability	Vayena et al. (2018), Yu et al. (2018) and Zeng et al. (2019)	It should be ensured that the use of AI in hospitals is understandable to the patient
	Transparency	Cath (2018), Vayena et al. (2018), Zeng et al. (2019), Froomkin et al. (2019) and He et al. 2019)	The visibility of the general logic of machine learning algorithms and its explanation should be ensured
	Trustworthiness	Yu and Kohane (2019)	It should be ensured that the patients and the physicians who use AI trust the systems’ predictions

One of the most mentioned ethical principles for using AI in healthcare was the principle of transparency (Cath 2018; Vayena et al. 2018; Zeng et al. 2019; Froomkin et al. 2019; He et al. 2019). It describes the visibility of the general logic of machine learning algorithms (Vayena et al. 2018). On the one hand, it is intertwined with the principles of explainability and explicability**,** which aim to not only make the algorithms transparent but also provide information for people with less technical knowledge such as patients or doctors (Cath 2018). Moreover, it seemed that explainability and transparency overlap and relate to similar issues. However, main difference between transparency and explainability is that transparency does not necessarily include further instructions such as a tutorial on how AI executes certain processes. If a hospital would provide access to the code of a system, they would provide transparency for this code; but to provide explainability, the code would need to be delivered with further explanation of its purpose and process. On the other hand, transparency is intertwined with the principle of fairness (Zeng et al. 2019). Zeng et al. (2019) stated that people in the context of healthcare might ask for transparency regarding the decision-making process of an AI out of concerns about fairness. However, we found no clear definition of what exactly fairness would mean in terms of AI and algorithms. We identified indications that in most fundamental manuscripts, the authors understand fairness as algorithmic fairness that ensures that there is no discrimination of minorities (Cath 2018). The results of the expert interviews confirmed the major relevance of transparency as an ethical principle. This especially refers to disclosing to medical experts how AI derives certain results. One expert clarified “I don’t know if that is possible, but I should ideally understand what the AI is doing” (E4).

In addition to transparent communication about the presence of an AI, the liability must be clearly evident (Vayena et al. 2018). The principle of liability is closely linked to accountability and responsibility (Schiff and Borenstein 2016; Reddy et al. 2019). We summarized those three terms using responsibility as it was the most frequent and interchangeably used term within the considered literature. Accountability for errors that occur through AI use in hospitals has not yet been conclusively determined. One interviewee compared this to the debate on self-driving cars: “This reminds me of the debate about self-driving cars. It is unclear who is responsible. The car manufacturer? The insurance company? The software manufacturer? The driver? This has not yet been conclusively clarified with regard to AI in hospitals either” (E6). Liability can be defined as the legally obligated determination of who is morally responsible for medical errors regarding the use of AI (Schiff and Borenstein 2016). While liability tends to address the legal aspects, accountability is more focused on the authority to issue instructions. Responsibility, on the other hand, includes an ethical and social component and addresses the questions of how much indirect responsibility is relevant and which actors are indirectly responsible. However, liability, responsibility, and accountability are not clearly delineated in most of the fundamental works and need further definitions, clarifications, and delimitations (Reddy et al. 2019). While the terms are often used synonymously, they can also sometimes be used too narrowly. In a case study, Luxton (2019) examined the ethical, responsible, and legal liability issues surrounding the use of IBM Watson in hospitals. They provided a guide for physicians who want to use AI tools in hospitals and identified precautions based on a case where patients with leukemia should be treated. The interviews revealed that while AI can be helpful in making suggestions, medical experts should be responsible for health-related decisions. One expert summarized “the human emotional aspects are simply missing. AI simply cannot consider every human aspect” (E3). Another expert added that “physicians possess numerous years of experience. Subjective human impressions might positively influence the treatment. There is still quite some information that an AI does not or cannot have.” (E6). Mentioned examples included the family background or health insurance.

Another reason why transparency regarding how algorithms work is highly ethically relevant is that the training dataset of an AI can influence the system’s output (Parikh et al. 2019). That means that algorithms trained on a specific group of patients (e.g., in a specific clinic of one city) may not be generalizable and interoperable. Therefore, when using AI in hospitals, generalizability should be ensured (He et al. 2019; McKinney et al. 2020) to avoid unintended outcomes that could potentially harm patients’ health. If an AI is too specialized on one task in one environment, it could deliver wrong treatment assistance when being transferred to another context. Generalizability could in this case be ensured if an AI would be tested in a multiple-case study.

When using AI in healthcare, most authors mentioned the avoidance of bias and harms as an important principle for physicians (Cath 2018; Char et al. 2018; Parikh et al. 2019; Reddy et al. 2019; Yu and Kohane 2019). Schiff and Borenstein (2016) discussed potential harms emerging from interactions between humans and AI when AI is considered as part of a medical team. They specifically discussed how responsibility should be distributed among physicians, developers, and further stakeholders, and they further provided advice for practitioners. Overall, we did not find much information or guidance on what exactly is possible harm and which precautions could be taken to avoid harm to patients. What we found was that education of an AI-literate workforce would play an important role when deploying an AI in a clinical environment (He et al. 2019). The introduction of an AI should therefore always involve all affected stakeholders, and all junior physicians need to be trained and educated in the areas of medical computer science and statistics (He et al. 2019). One expert explained, “I think especially young or unexperienced doctors benefit or learn from AI-based decisions. Experienced physicians have the most important parameters for the evaluation of certain disease in their heads, but this does not apply to novice physicians” (E6). In addition, the output of a predictive AI system in a health context should provide guidance for concrete medical interventions to explain the output of the prediction to physicians (Parikh et al. 2019).

One specific type of harm that was discussed in the fundamental articles was potential privacy issues (Cath 2018; Vayena et al. 2018; Zeng et al. 2019; He et al. 2019). However, we neither found detailed information on what exactly are the relevant privacy issues regarding AI use in healthcare, nor information on how possible issues could be addressed. One example could be an AI asking for sensible information that patients do not want to reveal.

When patients need to consent to the use of AI for a treatment or a therapy, they need to have trust in the system and the controlling physicians (Yu and Kohane 2019). Trust could be achieved through a high level of transparency and explainability. One important principle, related to transparency and explainability, is the informed consent process (Schiff and Borenstein 2016; Ting et al. 2017; Froomkin et al. 2019). To be able to agree to informed consent, the patient must understand how an AI is used and what consequences the use of an AI might have (e.g., on a treatment). Patients thus must to be made aware of the fact that some kind of AI is involved in their treatment or course of disease. One expert testified “In principle, the patient must agree to be ‘treated’ by an AI. This also implies explaining what this technology is doing and related consequences” (E6). This can be complicated for several reasons (Schiff and Borenstein 2016). First, the physician must have sufficient knowledge to explain the use of AI. Second, it is often difficult even for experts to understand the exact procedure of AI (black-box problem), since very large amounts of data and computing capacity are involved. One expert highlighted “We already heavily rely on certain technology. AI might yield in thinking less thus being less involved and losing the feeling of being responsible” (E2). Strategies to counteract this process could not be found in literature and need to be further investigated.

Yu and Kohane (2019) argued that the data and the algorithms need to be frequently controlled and updated to address the clinical workflow disruption. This requires not only the possibility of checking and updating, but also a continuous vigilance by the responsible physicians in hospitals (Yu et al. 2018). Not only does the system need to be checked and updated, but the feasibility of using AI in hospitals should be regularly updated as well (Gulshan et al. 2016; Yu et al. 2018; McKinney et al. 2020). It should be determined how exactly the use of AI would lead to an improvement in care (Gulshan et al. 2016). If the system is determined feasible and beneficial, the AI also needs to be checked for security issues to avoid cyber-attacks and errors (Zeng et al. 2019). Cyber-attacks could result in privacy violations, data misuse and even physical harm of patients through data and system manipulations.

We provide an overview of the ethical principles we extracted from the 15 fundamental manuscripts in Table 3. As not all principles were described in detail, we added some aspects of our understanding in the descriptions. Some principles were used interchangeably, which is why we provided just one description for up to three principles in some cases. We categorized the principles according to the types of issues that they may address. By regulatory issues, we refer to ethical issues that require clear rules and possible legal guidance, such as determining who is responsible for errors made by AI-assisted treatment. Normative issues are those that cannot be clearly defined by rules and laws, but should be guided by social norms (e.g., which patients should be treated first). As technical issues, we consider all types of issues that are caused by design (mostly unintentionally), such as a biased training dataset. Organizational issues are problems that could be addressed by restructuring processes within a hospital such as a lack of technical expertise of physicians, which could result in not being able to explain an AI-based treatment assistant.

In addition to the relationships between the ethical principles of AI discussed within the 15 fundamental manuscripts, we identified the citation structures between the articles. We found that the citations within our identified discourse ecosystem often differed from the citations of an article on Google Scholar or meta-databases such as Scopus meaning that the most cited manuscripts on these databased were not the ones that centrally discussed on ethical issues of using AI in hospitals. This highlights the importance of this modified discourse approach. The time span of the manuscripts we considered relevant for the ethical discourse on AI in hospitals ranged from 2016 to 2020. Most articles we identified were published in 2019. Ten of the articles formed a citation network, whereas five of the articles did not cite or were not cited by any of the other manuscripts. The most cited article within the identified network was also the most cited article on Google Scholar and Scopus on the topic of ethical frameworks of AI within healthcare. The most cited article within our identified papers was an empirical work and did not focus on theorizing on ethics and AI in hospitals (Gulshan et al. 2016). However, its findings, mentioned limitations, and conclusions were often used as a starting point for ethical discussions. In Fig. 2, we provide a timely overview of how the fundamental manuscripts cited each other and visualize ways in which future research could contribute to this network by referring to these valuable articles and connecting them to a holistic picture. For each fundamental article, we present the Google Scholar citations and the score in our network. The arrows symbolize a citation within the network and the dotted arrows offer possible points of reference for future research. Although some of the manuscripts cited each other, we found no article that discussed the others in light of ethical challenges and problems in hospitals. Rather, the articles often used different terms to describe similar aspects without referring to each other and did not specify important aspects.

## Discussion

Applying the modified discourse approach proposed by Larsen et al. (2019), we identified 15 manuscripts that are fundamental for the discourse on the ethical dimensions of using AI in hospitals. Although AI and healthcare are important application fields in many disciplines, we did not find one discipline that clearly stood out. Furthermore, the identified manuscripts made little reference to each other (see Fig. 2). Although we found papers such as Gulshan et al. (2016), which were cited more frequently among the fundamental manuscripts, these were empirical papers rather than contributions to the ethical discourse in the use of AI in hospitals. However, in our identified network, we could not detect any established work reflecting the current discourse in academia or considering the opinions of physicians with regard to ethical considerations and dimensions of AI. With this work, we address this issue (RQ 1). In addition, we provide a research agenda in the next chapter that aims to guide academia in future works (RQ 2).

We also found that the discourse did not followed a logical structure. Five articles we considered did not refer to any other manuscripts that we classified as fundamental (Cath 2018; Vayena et al. 2018; Zeng et al. 2019; Luxton 2019; Reddy et al. 2019). This could lead to parallel discussion streams on the same topic. Interestingly, the most cited manuscript among the fundamental manuscripts was an empirical work that addressed ethical dimensions in a limited way and only within the conclusion and limitations (Gulshan et al. 2016).

Most identified articles either provided an incomplete view of the ethical challenges of applying AI in hospitals or functioned as empirical works that just scratched the surface of ethical principles and issues. Some of the existing articles focused on ethical challenges of very narrow AI technologies and did not consider a bigger picture (Gulshan et al. 2016; Ting et al. 2017; McKinney et al. 2020). On the other hand, some of the articles tried to derive ethical principles for the use of AI in healthcare which did not really differ from general ethical principles for using AI (Cath 2018; Vayena et al. 2018; Zeng et al. 2019).

Considering the fundamental manuscripts, no article focused on an overarching moral principle such as virtue ethics. Rather, the ethical perspective was not clearly defined. In the context of the ethical use of AI in hospitals, this could be deeply problematic, as virtues can be used to provide guidance to an AI-based system about what is right and wrong (Siau and Wang 2020). Future research needs to build on ethical perspectives similar to how moral virtues are discussed by Beauchamp and Childress (2019) and transfer these considerations to the context of AI applications in hospitals. Our research aims to guide this process.

**Fig. 3:**
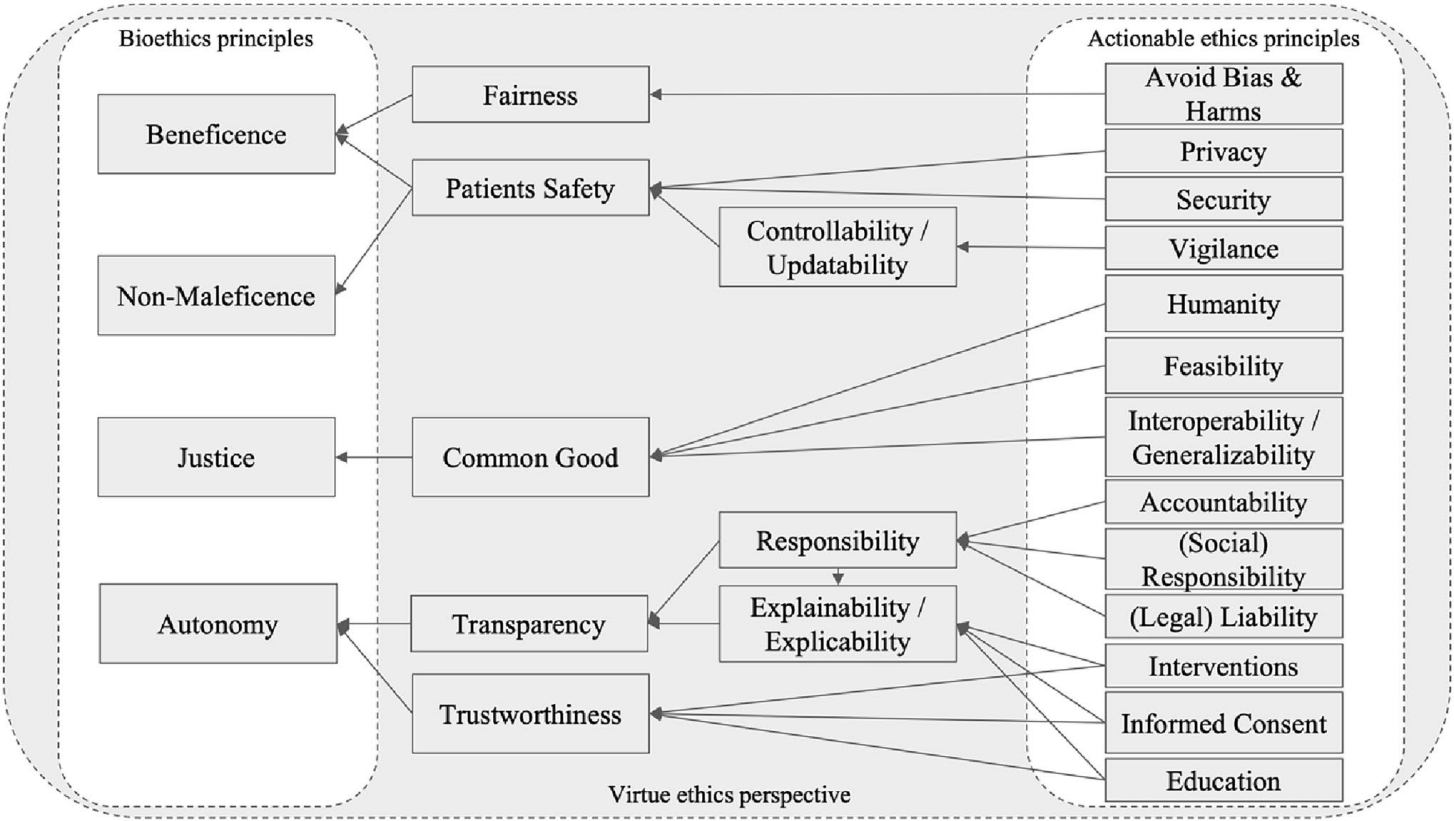
Visualization of the relationship between actionable ethical principles for using AI in hospitals and bioethical principles according to Beauchamp and Childress (2019) and Floridi et al. (2018)

Most of the principles we found were not discussed in detail and did not address the actual use of AI in hospitals (Char et al. 2018). In many articles, the same aspect was discussed using different terms such as explicability and explainability (Floridi et al. 2018; Vayena et al. 2018; Yu et al. 2018; Zeng et al. 2019) or accountability (Cath 2018; Vayena et al. 2018; Zeng et al. 2019; Reddy et al. 2019), responsibility (Cath 2018; Char et al. 2018; Zeng et al. 2019; Luxton 2019) and liability (Schiff and Borenstein 2016; Vayena et al. 2018; Yu et al. 2018; Luxton 2019; Reddy et al. 2019). In addition, ethics principles for using AI in healthcare are often intertwined and cannot be considered separately. However, we hardly found any discussion regarding dependencies between principles. Furthermore, detailed explanations on how ethical principles can be defined in the context of AI in hospitals were limited. Most principles lacked further definitions or were described on a meta-level that did not take into account ways in which they could be applied in healthcare. We, therefore, provide knowledge on how the principles should be examined and extended in future research. In Fig. 3, we show a structure that is more applicable for further research with dependencies of different levels of ethical principles for the use of AI in hospitals. Based on the relationships between ethical principles in the context of AI in hospitals, we provide a research agenda for academia.

## A research agenda for academia

A philosophical perspective that specifically addresses ethical dimensions of AI in hospitals does not appear in the current discourse; although it cannot be dismissed that individual papers exist that address this topic. Researchers from various disciplines need to include this ethical perspective in their future work, as philosophical venues are classically the drivers of ethical discussions. Within the identified manuscripts, we found different categorizations of ethical principles for AI. For ethical dimensions of using AI in hospitals, however, we could not find a common understanding of how to structure ethical principles. Therefore, we propose a research agenda for academia whose structure is based on the widely known articles from Beauchamp and Childress (2019) on biomedical ethics and Floridi et al. (2018), who applied these principles to provide an ethical framework for a moral AI society. We argue that although the same categories of biomedical ethics are relevant for considering ethical dimensions of using AI in hospitals, their definition and compliance are not clearly actionable in further research nor in medical practice. As an overarching moral principle, we focused on a virtue ethics perspective as suggested by Siau and Wang (2020).

With our research agenda, highlighted with the results from the expert interviews, we aim to guide future research to ensure that researchers theorize and discuss the most important issues and challenges of using AI in hospitals. With their knowledge, interdisciplinary scholars will be able to provide guidance for physicians who must make the decisions about the use of AI in hospitals. On the other hand, they can also ensure that AI is used by hospitals for the benefit of patients and not in the interests of, for example, hospital profitability. Based on the suggestions of Beauchamp and Childress (Beauchamp and Childress 2019), we structured our research agenda into the categories of beneficence, non-maleficence, justice, and autonomy. Future research can address either one of these categories or one of the four issue types from Table 3. For more applied work, we recommend addressing the issue types; for theoretical and philosophical work, we recommend addressing the categories of bioethical principles.

To provide guidance for future research, we propose the following research questions (Table 4), which are structured according to the four bioethical principles (Beauchamp and Childress 2019).

**Table 4:** Formal grouping of research questions to guide future research on ethical dimensions of AI in hospitals

Bioethical principles	Actionable principles	Exemplary research questions
Beneficence	Vigilance Security Privacy Avoid bias and harms	1. How can the principle of fairness be defined in the context of using AI in hospitals? 2. Which medical data should be used to derive AI recommendations for therapeutic and treatment processes? 3. How can AI systems inform decisions made by healthcare professionals? 4. How can disadvantages to patients belonging to certain minority groups be removed or reduced? 5. In which application domains of digital health can AI be introduced as decision support systems to enhance hospital procedures and patient treatment? 6. To what extent can AI assist with difficult therapy decisions for certain patient groups?
Non-maleficence	Privacy Security Vigilance	1. What are possible harms caused using AI in hospitals? 2. How can bias within the medical data used by AI be recognized and resolved by healthcare professionals? 3. How could a control mechanism for decision support for physicians through AI in hospitals be designed and developed? 4. How can the awareness of vigilance regarding AI used in hospitals be increased? 5. How can it be ensured that medical information is not retrieved by third parties? 6. To what extent can external data manipulations within AI datasets be detected and prevented by physicians?
Justice	Humanity Feasibility Interoperability/generalizability	1. How can AI applications in hospitals contribute to the common good of a society? 2. How can common good be defined and interpreted by AI applied in clinical environments? 3. Which guidelines are essential to ensure common good when using AI in hospitals? 4. To what extent can physicians be psychologically relieved of moral dilemmas when using AI in hospitals? 5. How is AI able to improve the doctor-patient relationship in hospitals? 6. How can existing AI applications in hospitals be transferred to other conditions, departments, countries, and cultures? 7. To what extent are generalizable AI results ensured?
Autonomy	Accountability (Social) Responsibility (Legal) Liability Interventions Informed consent Education	1. To what extent do physicians perceive themselves to be losing their autonomy when AI is applied in hospitals? 2. How should the application of AI in hospitals be transparently presented to medical experts and patients? 3. Who can be held accountable and socially responsible for AI-driven decisions, and under which clinical conditions? 4. How can the legal liability for using AI in hospitals be clarified and implemented in a legal foundation? 5. Who is accountable and responsible for ensuring legal alignment when using AI in hospitals? 6. How can AI accompany its outputs with concrete recommendations for use in medical interventions? 7. How can it be ensured that both the physicians and the patients are aware of the consequences when consenting to the use of AI in a hospital? 8. How should AI applications be designed to be utilized only under voluntary conditions among clinicians and patients? 9. How do we need to educate and train physicians to ensure an ethical use of AI in hospitals? 10. What kind of training increases trustworthiness in using AI in hospitals?

### Beneficence

Floridi et al. (2018) defined beneficence as a principle that ensures that an AI promotes the well-being of humans and its output favors the common good. But what does this mean in the context of using AI in hospitals? While AI should act in a fair way (Cath 2018; Vayena et al. 2018; Zeng et al. 2019), it is not clear exactly what this implies. Further research should address in more detail the aspect of fairness in the field of AI implementation in hospitals. This would ensure the beneficence of the system in favor of the patients. Fairness can be achieved by avoiding bias and harm to all patients. For example, the use of AI should not exclude certain minority groups (e.g., people with rare diseases). One expert emphasized, “There are also ethical differences within cultures. In some countries, abortion is simply not an option for women” (E1). Previous research has highlighted cases where AI delivered poor predictions in healthcare due to biased data (Vayena et al. 2018). There are data sources that do not represent the true epidemiology within a given demographic, for example in population data biased by the entrenched overdiagnosis of schizophrenia in African Americans. In this cases AI needs mechanisms to detect incomplete or biased data. However, research on this is rare. Although some studies have detected unfair behavior of AI in hospitals, limited research has been conducted on the prevention of such issues. Using rich dataset training data for an AI could be one approach to avoid unfairness in hospitals; but how this can be achieved is a question that should be addressed. The same applies for AI violating patients’ safety. Previous research has stated that patients’ safety is an important factor for deciding whether an AI-based system can be used or not (Char et al. 2018; Zeng et al. 2019; He et al. 2019; Parikh et al. 2019) and discussed cases where it was violated. However, research on how to ensure patients’ safety when subjected to AI treatment assistants is still rare. One expert underlined that, “AI should support with difficult therapy decisions securing the well-being of patients, for example, whether palliative or radiation treatment is more appropriate” (E3).

### Non-maleficence

AI use in hospitals should also be non-maleficent (Floridi et al. 2018). In contrast to beneficence, which includes what an AI should do, the principle of non-maleficence aims to avoid ethical issues when using AI e.g., in hospitals. However, in previous research, we did not find a comprehensive picture of the spectrum of possible maleficence caused by AI in hospitals. Due to the black-box character of AI, it is almost impossible to predict all consequences of its use, but the current state of knowledge could be depicted. It also remains unclear how non-maleficence in hospitals can be ensured when using AI. We could derive the following aspects from the literature that refer to non-maleficence: patients’ safety, privacy, security, controllability, updatability, and vigilance (Cath 2018; Char et al. 2018; Vayena et al. 2018; Yu et al. 2018; Zeng et al. 2019; He et al. 2019; Parikh et al. 2019; Yu and Kohane 2019; McKinney et al. 2020).

When applying AI in a hospital, possible violations of patients’ privacy must be identified and solutions need to be developed. However, AI could also cause physical damage to patients’ health, for example, when delivering decision support for diagnoses or medications. Although the highlighted training dataset is also potentially relevant for this, future research needs to determine which decisions could be supported by AI and how this decision support could be controlled. However, it seems that the decision support, e.g., regarding treatment recommendations, should always be monitored and assessed by human physicians: “It will never be the case that an AI takes over the complete diagnosis. It will always be the case that there is a choice and the human being decides at the end of the day” (E6). The technical controllability and updatability of a system, as well as the vigilance of the physicians, need to be ensured. In addition to monitoring AI for internal errors, we identified ethical issues regarding the external security of a system. For example, cyber-attacks could manipulate the data basis of an AI without the users noticing. Therefore, future research needs to address these types of security issues when using AI in hospitals. This leads us to the following further research questions: How can awareness for vigilance be increased?

### Justice

The principle of justice covers aspects that “contribute to global justice and equal access to the benefits” for individuals and society (Floridi et al. 2018). In the literature, we found overlaps with the principle of fairness that aimed at avoiding any type of discrimination (Cath 2018; Vayena et al. 2018; Zeng et al. 2019). For a sharper demarcation, however, in this article, we focus on the aspect of common good when mentioning fairness. Future research should investigate what common good exactly implies and how common good can be achieved by AI. This might contain “psychological relief from doctors in the context of a triage” (E2), i.e., classification of patients in a crisis according to the severity of the injuries, but also “improving the doctor-patient relationship when AI handles standard procedures” (E4). In the literature of fundamental manuscripts on the ethical dimensions of AI in hospitals, we found four actionable principles that can be assigned to common good and justice: humanity, feasibility, interoperability, and generalizability (Gulshan et al. 2016; Char et al. 2018; Yu et al. 2018; Zeng et al. 2019; He et al. 2019; Parikh et al. 2019; McKinney et al. 2020). Future research should investigate which AI applications in hospitals can benefit humanity. Furthermore, for each AI application, the technical feasibility of the application for the common good needs to be evaluated. In many cases, AI technologies in hospitals are only used for a very specific case within a system, e.g., in angiography: “There are AI-based systems, for example in angiography, which determine with a certain probability and based on certain points that are detected within a vessel, what the rest of the vessel might look like” (E6). Future research should focus on how to make these AI systems interoperable and how to make the outputs of an AI-based system in hospitals more generalizable.

### Autonomy

As another principle of bioethics, autonomy is defined as the right of patients to make decisions about their treatments, which implies that they mentally understand the situation (Beauchamp and Childress 2019). With AI, the question arises how patients’ autonomy can be ensured as we willingly “cede some of our decision-making power to machines” (Floridi et al. 2018, p. 698). Future research should focus on how autonomy has to be ensured when using AI as support for a treatment and how this autonomy can be achieved. One expert explained, “the patient is in the center of attention” (E1) and further “as a physician you cannot evade responsibility” (E4).

In the literature, we found two fundamental principles by which autonomy can be achieved: transparency (Cath 2018; Vayena et al. 2018; Zeng et al. 2019; Froomkin et al. 2019; He et al. 2019) and trustworthiness (Yu and Kohane 2019). If patients have transparency about the use and application of AI in a hospital on the one hand, and trust in the way it works on the other hand, autonomy can be achieved. One way of achieving trust is to “show the power behind it. If you do studies like the one with Watson and show comparatively that an AI achieves several times better results than a human expert, then that naturally creates trust” (E6). According to E6, presenting the advantages of accompanied studies could be an adequate strategy to increase trustworthiness. However, to ensure adherence to both principles, more detailed aspects must be considered. Transparency does not only imply that a patient is informed about whether AI is being used and could potentially understand how it works. Transparency also includes explaining to the patient exactly how an AI-based system works and how its use might affect his or her treatment (Vayena et al. 2018; Yu et al. 2018; Zeng et al. 2019). This requires considering not only the principle of explicability, but also the principle of responsibility. The patients must be aware of who is responsible for the consequences and outputs of the use of AI in a hospital. We found three types of responsibility that future research should examine more closely: functional accountability (Cath 2018; Vayena et al. 2018; Zeng et al. 2019; Reddy et al. 2019), social responsibility (Cath 2018; Char et al. 2018; Zeng et al. 2019; Luxton 2019), and legal liability (Schiff and Borenstein 2016; Vayena et al. 2018; Yu et al. 2018; Luxton 2019; Reddy et al. 2019). This is also in accordance with E6, who stated, “The question of responsibility has not yet been conclusively clarified and is, therefore, philosophical to a certain degree. We as company are accountable for keeping our stable clean. But we should also have the doctors who can also question this again in case of doubt. But a certain amount of legal liability should also lie with the manufacturer, who should also be responsible for ensuring that the AI is always up to date.”

Future research should, therefore, look at who is operationally responsible for AI and who has the authority to issue instructions on the use of AI, as well as who may not be directly responsible for the consequences of the use of AI but should be involved from an ethical perspective. In addition, it should be further investigated how the legal framework for the use of AI in hospitals should be designed and how it can be ensured that both physicians and patients are aware of it. A precise explanation of responsibility is part of the explainability of the ethical framework. How exactly this explainability can be ensured has not yet been sufficiently researched. We found three actionable principles that could enable explainability of AI: interventions (Parikh et al. 2019), informed consent (Schiff and Borenstein 2016; Ting et al. 2017; Froomkin et al. 2019), and education (He et al. 2019). Future research should address the fact that the use of AI should always be accompanied by concrete recommendations for interventions by physicians, as they must interpret the AI’s outputs. Further research is also needed to determine exactly how these interventions should be designed. Another sub-area of ethical research in AI is informed consent. Future research should explore ways to ensure that physicians explain the effects of the use of AI to patients well enough to enable confident decisions on whether to consent or refuse. However, to ensure explicability, physicians need to be trained in this matter. Future research should explore in more detail what types of training and education are needed to enable the explainability of AI to the patient. Interventions, informed consent, and education are also important components in creating trustworthiness. Future research should explore how exactly trust can be created in a system on the part of physicians and patients. However, trust in AI must be treated with caution, as clinicians “rely on the technology and become dependent on it” and further, “AI does the thinking and people act blindly” (E2).

## Conclusion and limitations

In this article, we presented the current discourse in the domain ecosystem of ethical considerations on AI in hospitals. Drawing from theoretical foundations (i.e., Beauchamp and Childress 2019; Floridi et al. 2018), enlightened by semi-structured expert interviews with clinicians, this article contributes to theoretical foundations by presenting research areas that need to be faced when AI is used in hospitals. These results are highly relevant for practitioners, academia, and healthcare researchers and inform societal issues and challenges.

The main theoretical contribution of this research is the proposal of a research agenda explaining where in-depth investigations are needed. Our study demonstrates that current research scratches the surface rather than conducting profound examinations. We thus guide scholars’ efforts for future studies and encourage the prospective discourse of ethical considerations of AI in healthcare. On a practical level, physicians comprehend to what extent the application of AI in hospitals seems fruitful as well as where ethical questions arise that could affect patients’ physical and psychological well-being. We, therefore, aim to raise practitioners’ awareness for the possible up- and the downsides of AI in healthcare. In terms of implications for society, individuals realize that ethical considerations of AI are vital, as the overall well-being of patients has the highest priority among clinicians.

As with all research, certain limitations apply. Since we aimed to identify highly relevant and fundamental theory-building papers (L1), we did not take a closer look at other papers citing these publications (L2, L3). In total, we have identified 15 fundamental articles, providing a sufficient foundation for our research agenda. However, it is possible that we could have missed some relevant literature investigating ethical considerations and dimensions of AI in hospitals, which may have provided additional knowledge. Moreover, we retrieved articles from interdisciplinary outlets and conducted a forward as well as backward search to obtain relevant publications from related disciplines. Even though the fundamental theory-building papers are from various disciplines and thus provide transferable results, publications from other sources (i.e., PubMed, an essential database for biomedical literature) might have yielded additional insights. Furthermore, the group of experts we interviewed was quite homogenous, with a small number of individuals that only cover a limited fraction of knowledge. Interviewing additional hospital employees, i.e., clinicians from other departments or employees working in other hierarchies as nursing staff, might have led to a more holistic picture.

We invite scholars to address the exemplary research questions we have provided in this article in the context of the bioethical principles. The citation network of the 15 fundamental manuscripts can be used as a starting point to better highlight the ethical discourse of AI in hospitals and to extend and deepen our discussion. We suggest that researchers consider virtue ethics as the main ethical perspective, as virtues need to be defined when AI-based systems are applied for treatment support in hospitals. The 18 ethical principles we found, and especially the 13 actionable principles, contribute to the discourse of AI use in hospitals and can serve as guidance for academia as well as physicians and healthcare decision-makers.

### Electronic supplementary material

Below is the link to the electronic supplementary material.

Supplementary file1 (DOCX 725 KB)

## Appendix

See Tables 5 and 6.

**Table 5:** Ranking of identified articles according to their number of citations

Number of citations	Number of papers
8	2
6	2
5	2
4	7
3	23
2	115
1	2713

**Table 6:** Interview guideline (German interview questions have been translated into English)

Phase	Research goal	Questions
Briefing	Welcoming the interviewee and providing general information about the research and brief introduction to the topic	–
Demographic data	Getting an understanding of the interviewee including position within the hospital and the areas of responsibility	a. Could you please introduce yourself? b. What is your current position in the hospital? c. What responsibilities does your position involve? d. How long have you been working in this position / in this hospital?
Ethical considerations in healthcare and hospitals	Ethical considerations physicians are confronted with and whether they follow a certain codex	a. What ethical considerations are you confronted with in your everyday work? b. What is the ethical code you follow?
Ethical considerations and technology	Ethical problems technology raises and how they are capable to resolve ethical issues	a. Which technologies are used in your hospital to support your work? b. Which technologies do you rely on for your decisions? c. Which ethical problems can technology cause? What questions arise? d. Which ethical problems can a technology help to solve?
Ethical considerations and AI	Specific questions on the application of AI in hospitals and which factors are crucial for a deployment and what ethical guidelines must be follow	a. What do you associate with the term “artificial intelligence”? Providing an explanation of AI and current examples to assume the same knowledge among all participants b. For which tasks can AI be used as support in hospitals? c. Which tasks can AI be allowed to take over independently and which not? d. Which factors must AI consider when being used hospitals? Which rules must be obeyed? e. What is AI not allowed to decide for itself? What outcomes need to be prevented? What negative consequences may result? f. What are ethical conditions, requirements, and challenges for the application of AI in hospitals? g. Which morally reprehensible decisions should AI not derive? h. Which moral decisions could an AI make better compared to a human being?
AI and future perspectives	Future ways of AI implementations in hospitals improving clinical procedures	a. For what purposes would you use like to use AI in hospitals? b. Which decision would you rather follow, that of a human or an AI? Please elaborate c. How do you think is the role of AI in hospitals changing in the future?
Debriefing	Debriefing of the interviewee and explanation of the research background, possibility for the interviewee to ask further question or giving closing remarks	a. What other question did you expect but was not asked? b. Do you have further questions / comments on the topic?
