# Supplementary material for: Artificial intelligence in hospitals: providing a status quo of ethical considerations in academia to guide future research
Source: AI Soc. 2021 Jun 28;37(4):1361–82. doi: 10.1007/s00146-021-01239-4 (PMC8238382; doi:10.1007/s00146-021-01239-4)

**Electronic Figure Submission**

Artificial Intelligence in Hospitals – Providing a Status Quo of Ethical Considerations in Academia to Guide Future Research


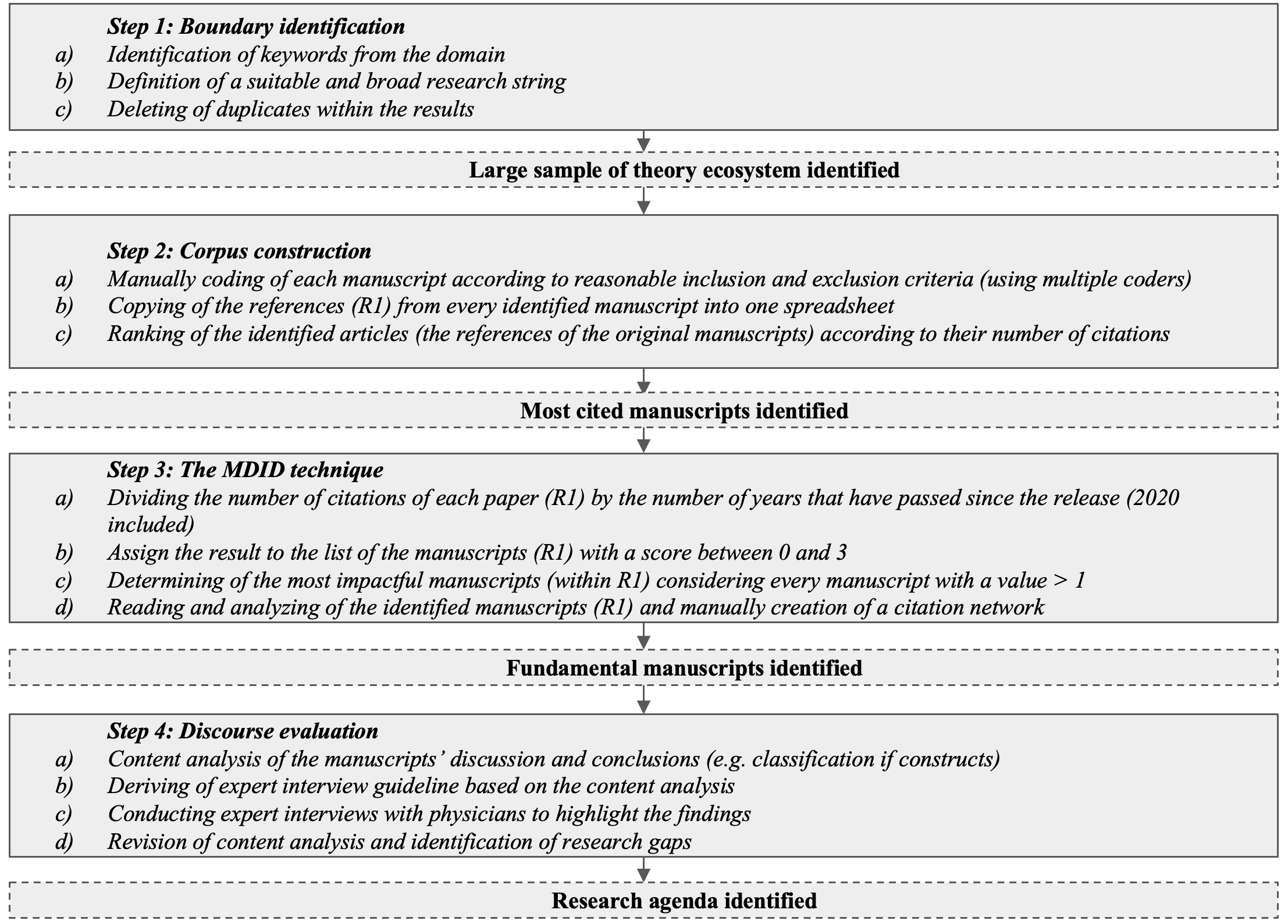


**Fig. 1** Adapted discourse approach based on Larsen et al. (2019) to derive a research agenda

Graphics program: Google Slides


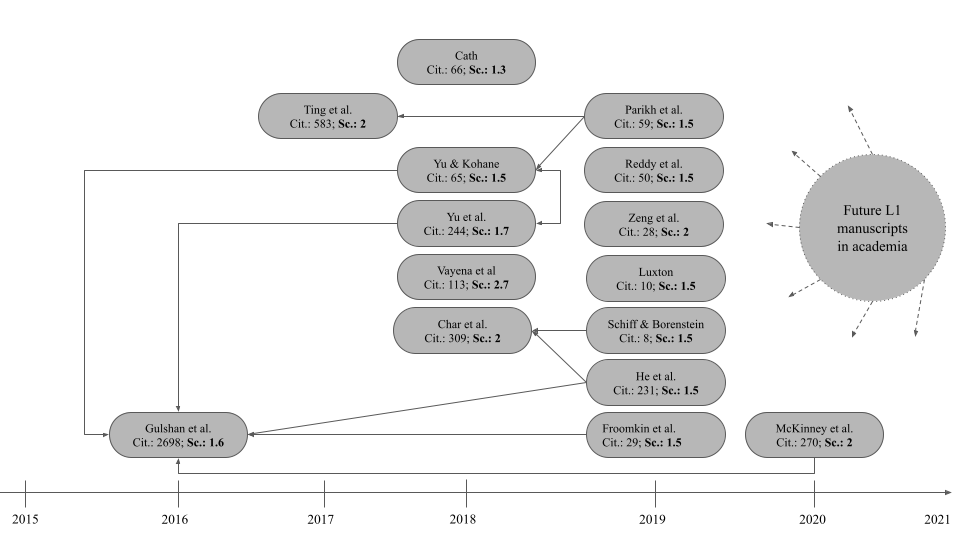


**Fig. 2** Citation network of the 15 fundamental manuscripts

Graphics program: Google Slides

**Fig. 3** Visualization of the relationship between actionable ethical principles for using AI in hospitals and bioethical principles according to Beauchamp and Childress (2019) and Floridi et al. (2018)

Graphics program: MS PowerPoint


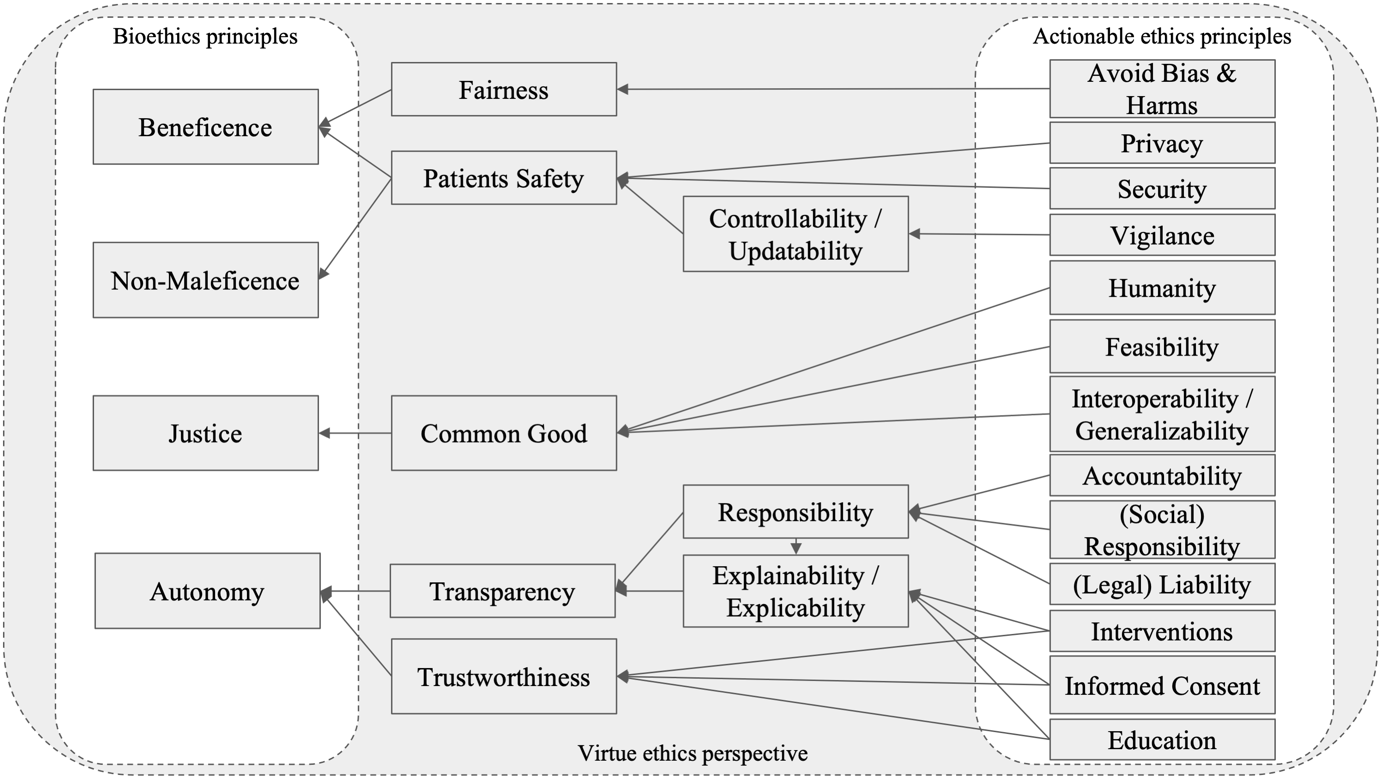

Supplement: Supplementary file 1 — Supplementary file1 (DOCX 725 KB) [file 146_2021_1239_MOESM1_ESM.docx]
